# Digital versus conventional techniques for construction of mandibular implant retained overdenture

**DOI:** 10.1186/s12903-025-05918-2

**Published:** 2025-05-05

**Authors:** Abd Elsalam Awad Ali, Ahmed Habib, Mohamed Shady

**Affiliations:** Prosthodontics Department, Faculty of Dentistry, Mansoura University, El Gomhoria street, Mansoura, 35516 Egypt

**Keywords:** CAD-CAM, Implant, 3D-Printed overdenture, Locator attachment, Occlusense, Superimposition, Occlusal wear, Occlusal force distribution

## Abstract

**Background:**

This study aimed to evaluate two different construction techniques (conventional and 3D-printed techniques) for two implant-retained complete mandibular overdentures regarding mechanical wear of the occlusal surface using 3D digital analysis and occlusion force distribution using the digital occlusal analysis system (Occlusense).

**Methods:**

Twenty patients were selected for this study from the prosthetic department, Faculty of Dentistry, Mansoura University. Each patient received two implants in the mandibular canine areas with locator attachments to retain the overdentures. According to the overdenture construction technique, all patients were randomly divided into two equal groups: the conventional heat polymerized overdenture group and the 3D-printed overdenture group. The mandibular overdentures were compared and evaluated immediately and after 12 months of insertion regarding mechanical wear of the occlusal surface using 3D digital analysis and occlusal force distribution using a digital occlusal analysis system (Occlusense).

**Results:**

3d printed group showed significantly higher occlusal surface wear than conventional group after twelve months (T12) with a p value ≤ 0.05. 3d printed group showed significantly more occlusal force distribution than conventional group with a p value ≤ 0.05. Also, there was a significant difference in occlusal force distribution with advance of time for each group, with a p value ≤ 0.05 level of significance.

**Conclusion:**

Within the limitations of this study, it was shown that implant overdentures constructed by 3D-printing techniques offer a promising results in distribution of occlusal forces for achieving occlusal equilibration. However, in term of wear resistance more developments need to be done to improve material properties.

**Trial registration:**

The study was approved by the local ethical committee of the Faculty of Dentistry, Mansoura University (No. A02060922) (6/9/2022) and retrospectively registered at ClinicalTrials.gov (ClinicalTrials.gov Identifier NCT06139003) (16/11/2023).

**Supplementary Information:**

The online version contains supplementary material available at 10.1186/s12903-025-05918-2.

## Introduction

For many years, conventional complete dentures have been a promising treatment for edentulous patients. They help restore a person’s appearance, speech, and normal oral function, which have a significant positive impact on people’s daily lives [1]. However, some problems have been reported with complete dentures in cases of resorbed ridge cases, contributing to biting, chewing abilities, facial esthetics and patient discomfort as a result of instability and decreased retention. Patients with edentulous mandibles now frequently choose implant-retained overdentures as a treatment option and their use has been the subject of substantial research even after [2].

According to the McGill and York consensus statement, a 2-implant supported mandibular overdenture should be the standard treatment for edentulous mandibular patients. Dental implants placed in the anterior mandibular region showed significant reduction in bone loss of the residual ridge, they also provide better retention and stability, as well as greater chewing comfort and patient satisfaction [3].

Other issues were reported with complete dentures related to the material and techniques of construction, such as lack of dimensional stability, changeability of color, and a reduced fit between the tissues supporting the denture and the base. Increased porosity in dentures affects their aesthetics and mechanical properties [4]. They also require complicated clinical and laboratory procedures [5].

CAD/CAM manufacturing is based on two main technologies: subtractive (milling) and additive (3D-printing) technologies. Subtractive (milling) manufacturing is used in dentistry, especially in prosthodontics. This technique involves the milling of complete denture prostheses from pre-polymerized manufactured blocks. However, waste materials and milling burs wear are seen as the primary drawbacks of CAD/CAM subtractive technology. So, efforts are being made to advance additive manufacturing or 3D-printing techniques for the construction of complete dentures, as the latter has demonstrated a significant degree of efficiency in reducing wasted materials. The additive (3D-printing) manufacturing technique fabricates complete denture prostheses from photopolymerized acrylate material using a 3D laser lithographic (LL) machine joining materials layer by layer in a sequential manner [6].

When complete removable dentures are made using 3D-printing instead of milling processes, the process is quicker, less expensive, and uses less material. Nevertheless, in comparison to milled materials, the mechanical and physical characteristics of materials used in 3D-printing processes remain subpar [7].

These digital dentures require fewer laboratory visits, potentially requiring just two appointments to complete the process. They also offer a more accurate base fit and greater retention than traditional heat-polymerized dentures. Encouraging short-term clinical performance, favorable patient-related outcomes, and appropriate time-cost effectiveness [8].

The occlusal goal of prosthodontics is to achieve bilateral balance of removable complete overdentures in order to address these issues. As digital technology has advanced, several occlusal force assessment devices have entered the dentistry market. With the use of a novel technology called Occlusense, clinicians can greatly improve the prosthetic occlusal balance upon placement by utilizing computer-guided occlusal force-finishing correction changes. It is now possible to tackle this clinical force balancing problem digitally [9].

Also wear resistance is a crucial physical characteristic of prosthetic teeth. It assesses the prosthesis’s capacity to uphold the established occlusal relationship, which makes it a significant factor in determining the longevity of dentures. Along with speech and aesthetics, patients have high expectations for the masticatory effectiveness of their new dentures, which may be hampered by denture tooth wear. Surface loss is typically more noticeable on posterior teeth than on anterior teeth. Denture teeth wear over time as a result of stresses of mastication during functional and parafunctional movement leading to the potential for lost occlusal vertical dimension, reduced masticatory efficiency, fatigue of the masticatory muscles, discomfort for patients and aesthetic concerns [10].

Although the field of prosthodontics seems to have undergone major change as a result of digital manufacturing dental technology, this creative field still requires further investigation. Thorough research is required to determine the long-term function of these digitally created dentures, the biocompatibility of the materials used, and the manufacturing accuracy of 3D printing. To fully realize the potential of 3D-printed dentures, clinical studies involving an array of patients and for prolonged periods of time are essential. Fit, efficiency, comfort, and durability should all be closely monitored throughout these trials and compared to the gold standard of traditional dentures. The purpose of this study was to compare conventional heat polymerized overdenture (group A) and 3D printed overdenture (group B) supported by two dental implants regarding mechanical wear of the occlusal surface using 3D digital analysis and occlusal force distribution using the digital occlusal analysis system (Occlusense). The hypothesis of this study would be a significant difference between two groups to be found regarding occlusal wear and occlusal force distribution.

## Materials and methods

### Study design

Twenty completely edentulous patients were selected from the Prosthetic Department, Faculty of Dentistry, Mansoura University. The patients were fully informed about the purpose as well as the surgical and prosthodontic treatments of this study. They signed the written consent of the Ethical Committee of Mansoura University Dental Research (No. A02060922). (Clinical trial registration number: NCT06139003) *(16/11/2023)*. According to the following criteria: a completely edentulous, healthy firm mucosa without any remaining roots or jaw cysts. Maxillomandibular Angel’s class I relationship with sufficient restorative space (minimum of 8.5 mm measured by using putty index). Sufficient residual alveolar bone quantity and quality class 1–3 according to Lekholm and Zarb [11]. The sample size was determined based on results from a previous clinical trial (effect size = 1.1, α = 0.05, β = 0.90) [12]. The calculated sample was 20. The power analysis was performed with the aid of computer software (G*power 3.1.5, Heinrich-Heine-Universität Düsseldorf, Germany ). Balanced randomization was used to equally assigned patients to one of two groups using to ensure comparability between groups. The participants were randomly assigned into two groups using random numbers generated in an Excel spreadsheet. The two groups are: conventional group and 3d printed group.

Patients with systemic issues such as recent myocardial infarction, hepatic patients, bleeding disorders, autoimmune diseases like rheumatoid arthritis, hyperparathyroidism, uncontrolled diabetes mellitus, severe osteoporosis or cancer were excluded from the study. Also, patients with recent organ transplants, long-term corticosteroid use, radiotherapy, uncooperative patients with psychiatric disorders, heavy smokers, poor oral hygiene, and parafunctional habits were excluded.

## Presurgical procedure

### Construction of complete dentures

Preliminary impressions of the upper and lower jaw were taken with irreversible hydrocolloid impression material (Cavex alginate, Cavex, Holland) in modified stock trays. After pouring in dental stone (Lab stone, Miles dental Product, Miles, INC, South Blend, USA.), customized trays had been constructed in autopolymerizing acrylic resin (Pekatray, Bayer. Dental, Lever Kusen, Germany). Green compound (Hiflex Thermoplastic impression green sticks, Prevest Denpro, India.) had been softened and employed to the custom trays with appropriate extension, then final impressions were obtained by applying zinc oxide eugenol free paste (Zinc Oxide Eugenol, Cavex, Holland). These impressions were then poured in dental stone (Lab stone, Miles dental Product, Miles, INC, South Blend, USA)to create master casts. Blocks were created to record the jaw relation, and a maxillary cast was mounted on a semi-adjustable articulator (Whip Mix Corp., Louisville, KY, USA) utilizing a face-bow transfer (Whip Mix Indirect Mounting Facebows model 9155). Centric, protrusive and lateral inter-occlusal data were then recorded to mount the mandibular casts, then arrangement of cross-linked acrylic artificial teeth following bilateral balanced lingualized occlusion (NT Unay acrylic resin teeth, Toros Dental, Turkey). Maxillary anatomic teeth were used opposing mandibular semi-anatomic teeth with reduced buccal cusps [13]. This arrangement allows for the maxillary palatal cusps to be the only cusps in contact, allowing for liberty of movements (long centric). Then the trial denture was meticulously sculpted using wax (Base plate Modeling wax, Cavex, Holland BV). Try in was done then the final denture was processed utilizing a long curing cycle.

Occlusal adjustment was carried out by using laboratory remount after deflasking. Using two colored articulating paper the maximum intercuspation, as well as lateral excursive and protrusive movements were refined by selective and spot grinding then the teeth were polished.

The dentures were delivered to the patient and then clinical remount was done. The occlusal adjustment was done by selective and spot grinding.

### Implant placement surgery

Under local anesthesia and antibiotic cover (1 gm. of clavulanic acid with amoxicillin 1 h before surgery). A crestal incision with the full thickness of the flap was made, the crestal bone was trimmed in cases with irregular alveolar bone crest to ensure that implants placed at the same level. Each patient received two implant fixtures (BTK dental implants) in the canine region of the mandible, and insertion torques ≥ 35 Ncm were acceptable, then covered immediately by the cover screw. Post-operative panoramic x-ray film was made to verify the position and orientation of implants. The lower denture was fitted with relief over the implant site to avoid loading the implants, and soft lining material was applied, with the occlusion refined.

### Prosthetic procedures

**Fig. 1:**
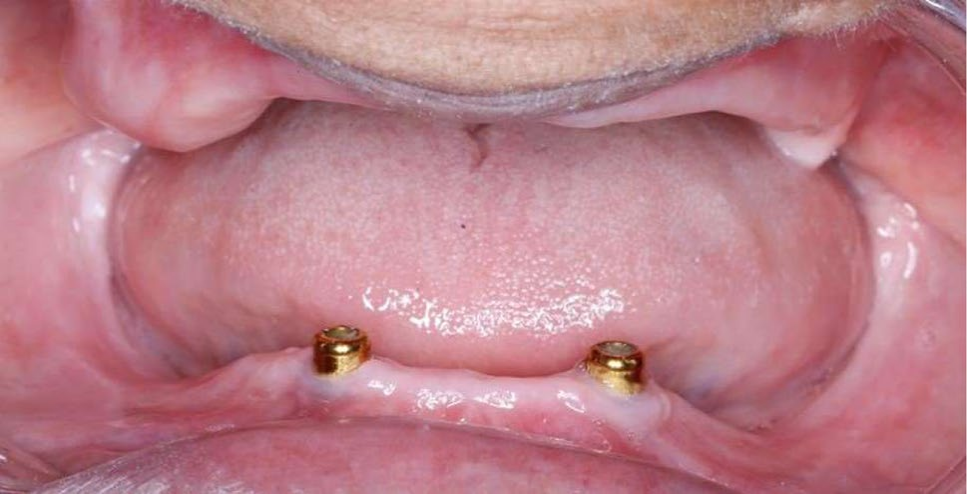
Locator attachments screwed to implants

After three months of osseointegration, implants were exposed using the tissue punch, and the healing abutments (BTK, KR H3.5 mm Ø4.5 mm ref no. 201KR3A1) were screwed over each fixture. After two weeks, the healing abutments were unscrewed, and locator abutment attachments (BTK dental implanting trust, Italia) (2.5 mm diameter and 4 mm height) were screwed to implants and tightened by the torque wrench (30 N/cm) (Fig. 1).

### Construction of a new complete overdenture

According to treatment, all patients were randomly divided into two equal groups:

Conventional heat polymerized overdenture group: patients received a maxillary complete denture opposing a mandibular implant overdenture constructed by conventional technique.

3D-printed overdenture group: patients received a maxillary complete denture opposing a mandibular implant overdenture constructed by 3D-printing.

### Construction of conventional heat polymerized overdentures

A metal housing was placed over locator abutments with a black lab processing insert. then construction was completed conventionally as mentioned before in conventional complete denture construction.

### Construction of a 3D-printed overdenture

#### Generation of a 3D-printed edentulous master cast

**Fig. 2:**
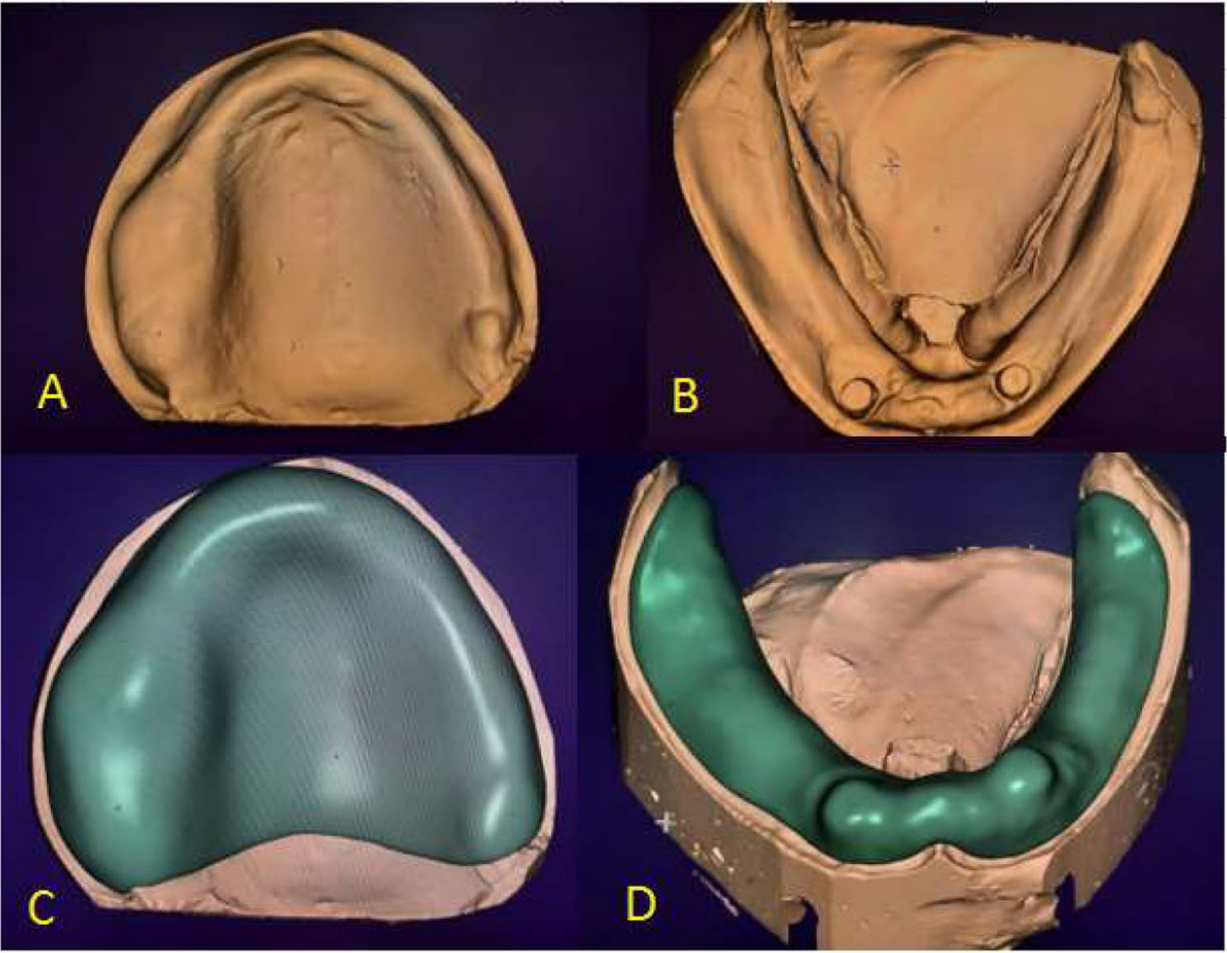
CAD design for record base construction

A metal housing (690NA022 Kit Locator^®^ Metal Cap + Blockout Spacer, BTK) was placed over locator abutments with a black lab processing insert. Mandibular and maxillary preliminary impressions were taken using an irreversible hydrocolloid impression material (Cavex alginate, Cavex, Holland). After pouring preliminary impressions, self-cure acrylic resin special trays were constructed, and then final impressions were taken using medium body addition silicone material (Thixoflex, M.zhermack.com, Italy), then poured, and master casts were obtained. The maxillary and mandibular master casts were scanned using a scanner (AnyScan, Vinyl HR, Germany), in three dimensions to produce a virtual master cast and saved as standard tessellation language (STL) (Figure 2a and b).

### Designing and printing of temporary record base

The virtual master cast STL file was imported into Exocad software (Exocad, GmbH, Darmstadt, Germany). to design the virtual record base, where the anatomic landmarks were identified and the peripheral limits were marked with a thickness greater than 2 mm extended to the vestibule area (Figure 2c and d). Then it was separated from the virtual master cast and oriented at a slight angle, in which the fitting surface was away from the build platform and supports were generated around the perimeter of the virtual record base. The tank of the 3D-printer (EPAX 3D 4K 6.6” printer, USA) was filled with temporary resin material (EPAX 3D, Temporary Printing Resin, China), afterwards, printing was started. After complete printing, the supports were removed, and the finishing and polishing were done by the conventional method.

### Fabrication of the occlusion rim

Wax occlusion rims were added to maxillary and mandibular printed record bases. intraorally, the labial fullness was established, the occlusal plane and occlusal vertical dimension were recorded, and the reference lines (midline, canine lines, and smile line) were marked with cuts in the wax on the facial surface of the wax record blocks. The centric, protrusive records were registered, and the lateral jaw relation record was calculated from the Hanau equation.

### Scanning and designing a virtual complete overdenture

**Fig. 3:**
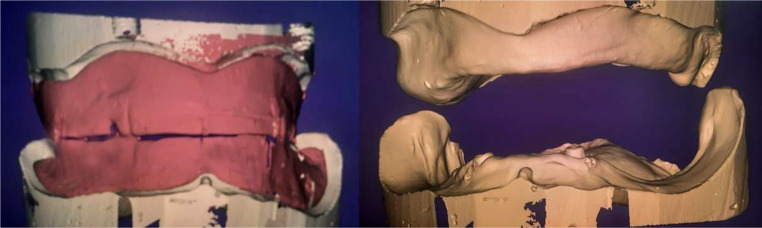
Digital maxillo-mandibular relationship

The record blocks were mounted on a semi-adjustable articulator in the laboratory, then scanned in a position close to the clinical situation using the same extra-orally scanner (AnyScan Vinyl HR, Germany), saved in an STL file, and imported into Exocad software, which is able to detect point-by-point matching of the anatomical landmarks on the virtual master casts and superimposed with virtual record blocks (Fig. 3). Different tooth library brands and shapes are included in the Exocad software. Then it automatically proposes a bilaterally balanced set-up. It is also possible to customize the set-up by modifying the position or morphology of one or more teeth or even removing them. Then the use of 30-degree teeth was set up as one unit according to jaw relations, and the occlusal plane, which was defined according to anatomical landmarks and reference lines, was determined. To achieve a natural look, the volumes and dimensions of the papilla, the marginal curve, the canine eminences, and the finishing can be adjusted. After a complete matching process of 3D virtual maxillary and mandibular master casts with a virtual complete overdenture, we obtained the position relationships of the maxillary and mandibular jaws and saved the virtual complete overdenture as an STL file.

### Fabrication of a 3D-printed trial overdenture

The denture base and teeth were designed with Exocad software as one solid piece, with the occlusal side facing toward the build platform, and supports were generated around the perimeter of the denture with added supports to the teeth. Then it was exported to the printer and printed with the same steps and the same temporary resin material. After printing, rinse, remove the supports, and finish and polish the printed denture using the same conventional method. After that, the trial overdentures were tried intra-orally. The maxillomandibular relationship, occlusion, lip support, and tooth arrangement were verified and confirmed by asking the patient if they were satisfied with their trial overdentures.

### Fabrication of a 3D-printed permanent overdenture

**Fig. 4:**
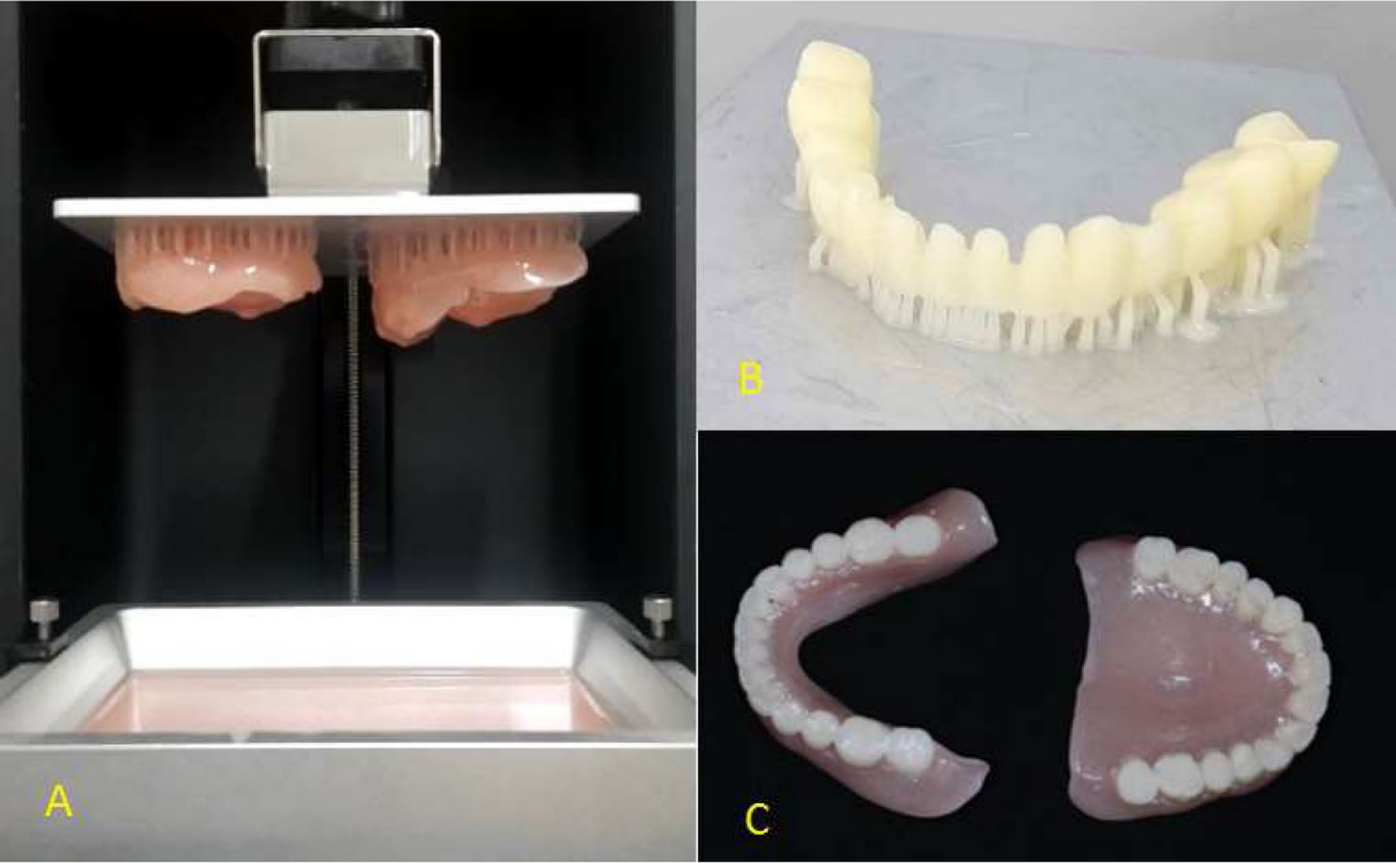
3D printing construction steps for definitive complete overdenture

For maxillary and mandibular dentures, the denture base and teeth were designed as separate files. The virtual denture base was oriented at a slight angle with the intaglio surface toward the build platform, and supports were generated around the perimeter of the virtual denture base with the desired slice thickness selected and removed from the denture tooth sockets and cervical margin. This file was then exported to the printer. The 3D-printer was filled with denture base resin (ifun, China), a shade was selected based on prescription, and the same method of printing was started. The virtual denture teeth were printed with the same method as one unit with white dental resin (Ifun, China), with the occlusal surface oriented toward the build platform. After the 3D-printed denture is completed, remove the printed denture base and teeth from the build platform and place them in a container filled with isopropyl alcohol (99.9%, Petrochem, Dubai) to rinse off the residual uncured resin, then wash and air drying to remove alcohol from the surfaces. After that, the remaining supports were removed from the base and teeth and finished by cutting a disc and rubber wheel and polishing with wet sand using a conventional method. For post-processing polymerization, the printed teeth were glued to the printed denture base, a small amount of denture base resin was applied to the tooth sockets, and the teeth were bonded while placed on the articulator, then placed in a post-curing unit (Mogassam, Egypt) for 20 min until the teeth were set in position, ensuring complete polymerization. Finally, the permanent complete overdentures were glazed by using a brush with nano-filled, light cure (Vita Akzent LC Glaze, USA) and cured by inserting them into the post-curing unit (Fig. 4).

The post-processing (curing) and addition of glaze to 3D printed dentures significantly improve their mechanical and aesthetic qualities, curing and glazing of 3D printed dentures involve several essential steps:

- Curing: After the dentures are printed using photopolymer resins, they need to be fully cured using UV light exposure. This step enhances the mechanical properties, ensuring the dentures are strong and durable.
- Cleaning: Once cured, it’s important to clean the dentures to remove any uncured resin. This can typically be done using isopropyl alcohol or a similar solvent.
- Glazing: This step involves applying a surface treatment using a brush with nano-filled, light cure (Vita Akzent LC Glaze, USA) to improve the aesthetic quality and provide a smoother finish that is subsequently cured.
- Final Adjustments: After the glazing process, any necessary adjustments for fit and comfort should be completed before the dentures are ready for use.

### Direct functional pick-up procedure

**Fig. 5:**
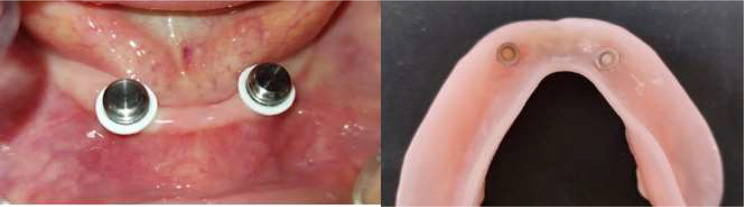
Direct functional pick-up of locator attachments

For all patients, relief was done on the fitting surface of the mandibular denture opposite to the locators to allow space for the self-cure acrylic resin with metal housing and ensure proper seating, as proved by the absence of rocking. Then small vent holes were prepared lingual to the site of attachment to allow exit for excess acrylic resin. The white locator blocking rings, which block the undercut, were stretched over the locator abutments, followed by pressing the metal housings with the black lab processing insert directly over the locator abutments. The denture was then dried, and the relieved areas were slightly coated with acrylic resin monomer. After that, self-cure acrylic resin was mixed according to manufacturer instructions, and a small amount was applied to the relived areas. The denture was then reseated into the patient’s mouth, and the patient was instructed to hold it in place in centric occlusion until complete polymerization of the acrylic resin material. The denture was removed; finishing and polishing of the denture was done, and the black inserts were replaced by pink nylon inserts (of moderate retention values) with the aid of the insertion tool (Fig. 5). The occlusion was readjusted if necessary to ensure proper occlusal contact.

### Evaluation of the mechanical wear of occlusal surfaces

**Fig. 6:**
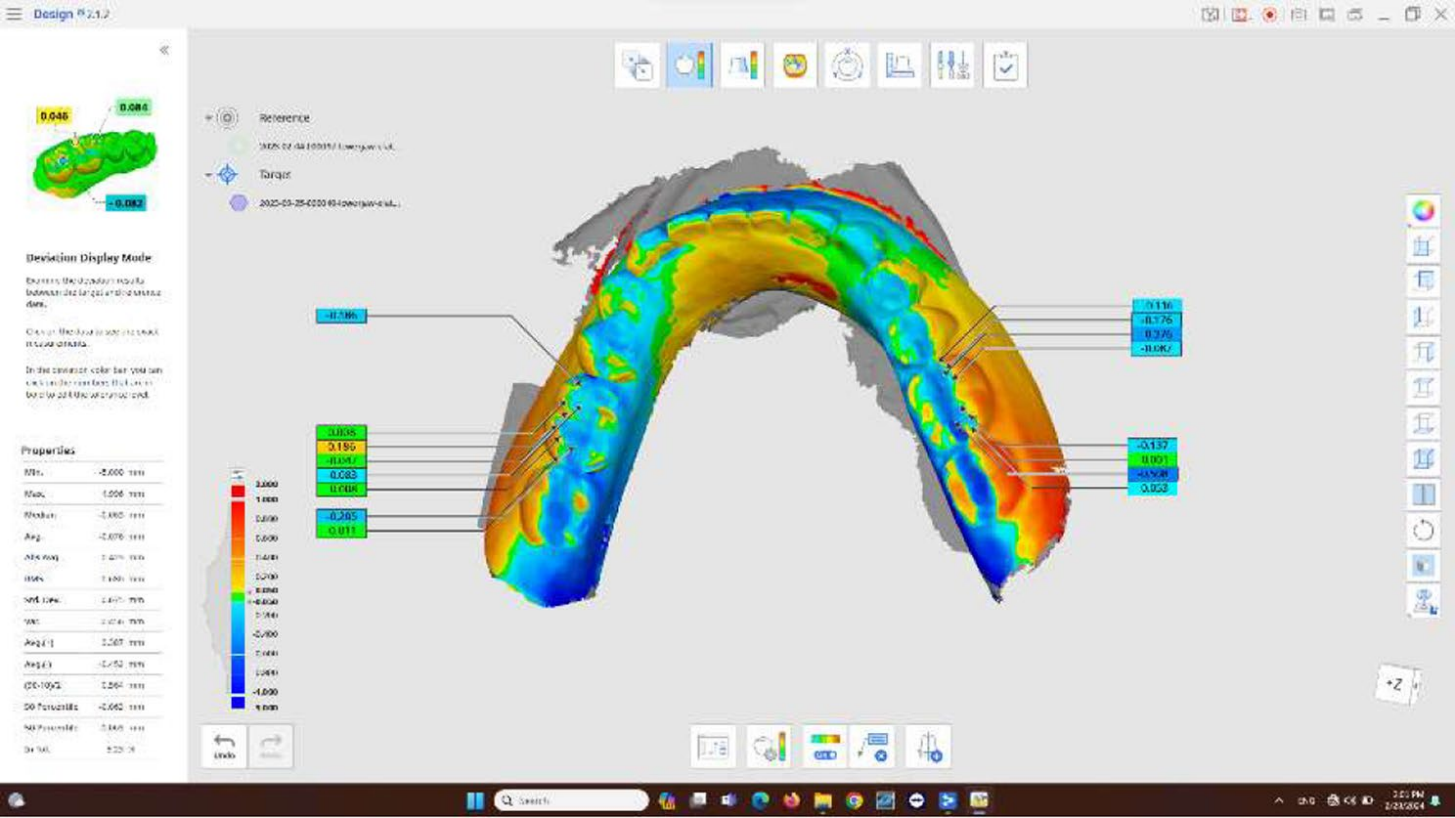
Measurement of occlusal wear using 3D digital analysis

The mandibular and maxillary dentures were scanned with an intraoral scanner (Medit i700, Korea) (Trueness 24.4 μm, Precision 21.4 μm) to create 3D virtual overdentures with all tooth surfaces recorded, then saved to STL files at two different periods: T0: at the time of overdentures being inserted, and T12: after twelve months from overdenture delivery, each pair of STL files were superimposed by using Medit Link application (v 3.0.4; Medit). The software automatic best-fit alignment was used to superimpose the pair scans (Trueness 78.35 μm) [14]. Then selected points on the occlusal surfaces of all teeth were assessed (three points on the mesial, middle, and distal incisal edges of anterior teeth and the cusp tip, cusp slopes, and cusp ridges of all posterior teeth) (Fig. 6). The deviations between the points were examined. The denture (T12) was compared to the baseline (T0) to determine the amount of wear encountered after twelve months of use.

### Evaluation of digital occlusal force distribution by occlusense system registration

Occlusense is a wireless digital system handle and sensor that sends data to an iPad application through a Wi/Fi connection, then the app shows the recordings of the patient. The handle is wide and triangular to accommodate the sensor tab, The handle of occlusense has a pink control button: run the daily function test and start and stop recording, Green control button: start sending data over Wi/fi, latch door at the top of the handle, which is opened to replace sensors and latches to hold the sensor in place while in use, a LED light that indicates whether the handle is charged. A LED display window shows setup, Wi/Fi connection and recording instructions, it also shows a progress bar of the recorded data processing. The Occlusense handle can record up to 0.056 s/frame of incremental digital occlusal data, it has to be charged beforehand in order to function, and it will shut off after four minutes of inactivity. A daily handle function test that uses a test sensor is necessary for calibration in order to determine whether the device is functioning as intended and the app will indicate whether the test was successful. The handle can record and send patient digital occlusal data to the iPad when the function test is over, the thickness of the occlusense sensor is 60 microns. Its edges are secured to a stiff cardboard frame that encloses the sensor’s metallic contacting parts. The cardboard frame has no aid for consistently positioning the sensor in a patient’s mouth [15]. 

**Fig. 7:**
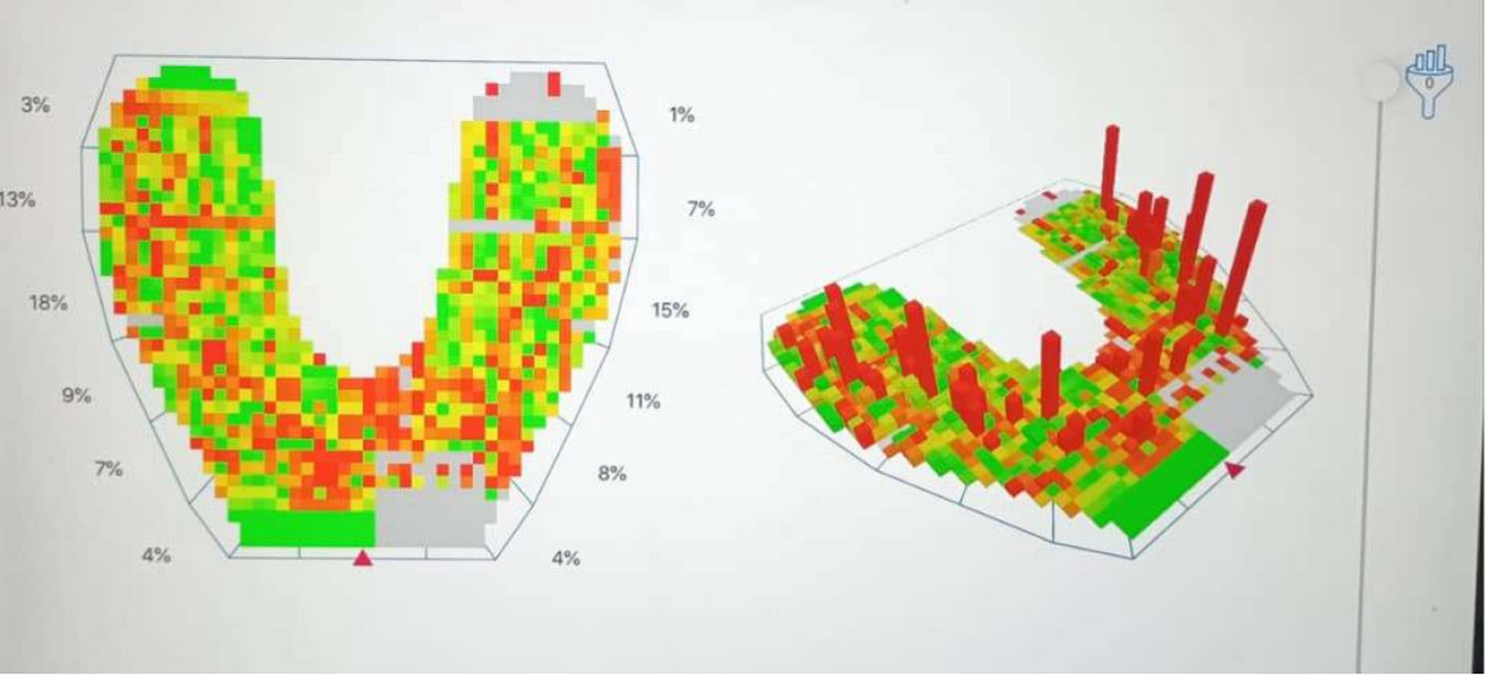
Measurement of occlusion stability using the digital occlusal analysis system (Occlusense)

The test sensor was inserted inside the handheld after connecting it to the same Wi-Fi connected to the iPad containing the occlusense software. After that, the sensor was removed from its packaging and inserted inside the handheld; the direction and position were determined by the markers on the sensor and by three positioning pins. An upright patient and sensor were inserted into the patient’s mouth, and the patient started to bite on the sensor according to the instructions of the operator. The pink button was pressed, and the occlusal state was recorded. The recording ended automatically, and the data was saved locally on the handheld, then transmitted to the iPad-App through a wireless connection. After that, the recording data was stored in the patient file in the iPad-app. The sensor was removed from the patient’s mouth. A graph of the data was then viewed in 2D, 3D, or in a combined view (Fig. 7). The sensor was removed from the handheld and disposed of properly after the desired recordings had been made. These recordings were saved at two different times: T0: at the time of overdentures being inserted, and T12: after twelve months from overdenture delivery.

## Results

### Statistical analysis

Data were analyzed using the Statistical Package of Social Science (SPSS) program for Windows (Standard version 26). The normality of the data was first tested with the Shapiro test.

Continuous variables were presented as mean ± SD (standard deviation) for normally distributed data. The two groups were compared with an independent t test, while paired groups were compared with a paired t test. The threshold of significance is fixed at the 5% level (p-value). The results were considered significant when the *p* ≤ 0.05. The smaller p-value obtained, the more significant the results.

**Table 1:** Comparison of occlusal wear between conventional heat polymerized overdentures and 3D-printed overdentures after twelve months (T12)

Occlusal wear	Conventional heat polymerized overdenture (*n* = 10) (mm)	3D-printed overdenture (*n* = 10) (mm)	Independent t-test		*P* value
Incisors	0.038 ± 0.01	0.144 ± 0.009		t = 16.44	0.001*
Premolars	0.075 ± 0.02	0.165 ± 0.009		t = 12.83	0.001*
Molars	0.061 ± 0.01	0.156 ± 0.011		t = 13.78	0.001*

*Significant *p* ≤ 0.05

Table 1 shows that 3D printed overdenture group showed significantly higher occlusal wear values than conventional group after twelve months (T12) at all areas with a p value ≤ 0.05.

**Table 2:** Comparison between conventional heat polymerized overdenture group and 3D-printed overdenture group regarding occlusal stability

Occlusal stability		conventional heat polymerized overdenture (*n* = 10) (%)	3D-printed overdenture (*n* = 10) (%)	Independent t-test	*P* value
Incisors	T0	14.80 ± 2.97	15.70 ± 3.59	t = 0.61	0.549
	T12	25.00 ± 3.49	20.50 ± 3.03	t = 3.07	0.006*
Premolars	T0	52.50 ± 3.13	55.00 ± 6.20	t = 1.14	0.270
	T12	46.40 ± 2.87	39.80 ± 3.73	t = 4.43	0.001*
Molars	T0	32.50 ± 4.14	29.30 ± 6.34	t = 1.34	0.198
	T12	28.70 ± 4.32	36.60 ± 3.83	t = 5.96	0.001*

*Significant *p* ≤ 0.05

Table 2 shows that there was an insignificant difference between both groups at the time of insertion (T0) with p value > 0.05 However, after twelve months (T12) 3d printed group showed significantly higher distribution in occlusal force than conventional group with a p value ≤ 0.05. With still uneven occlusion between incisors, premolars, and molars in both groups.

**Table 3:** Comparison of occlusal stability at different evaluation periods for each group

Occlusal stability	T0 %	T12 %	Paired t-test	*P* value
conventional heat polymerized overdenture				
Incisors	14.80 ± 2.97	25.00 ± 3.49	t = 9.24	0.001*
Premolars	52.50 ± 3.13	46.40 ± 2.87	t = 4.35	0.002*
Molars	32.50 ± 4.14	28.70 ± 4.32	t = 2.21	0.05*
3D-printed overdenture				
Incisors	15.70 ± 3.59	20.50 ± 3.03	t = 2.99	0.015*
Premolars	55.00 ± 6.20	39.80 ± 3.73	t = 5.51	0.001*
Molars	29.30 ± 6.34	36.60 ± 3.83	t = 3.89	0.004*

*Significant *p* ≤ 0.05

Table 3 shows that there was a significant difference in occlusal force distribution with advance of time for each group, with a p value ≤ 0.05 level of significance.

## Discussion

Non-guided surgery was chosen in this study over steriolithographic surgical guides. Since the surgical stent controls the entire process in fully-guided operations, Accuracy is therefore seen as the primary advantage. On the other hand, the template may obstruct the irrigant solution’s flow, which could raise the temperature, cause bone resorption, and harm the osseointegration process overall. The intended and actual placement of implants using mucosa-supported surgical guides differ more in clinical investigations. Because there are numerous crucial clinical steps between inserting the CAD/CAM surgical guide and the full seating of the implants, such as restricted mouth opening and challenging anchor pin securing, the overall precision may be compromised.Verhamme and colleagues used a particular kind of stereolithographic surgical guide to implant 72 implants in 12 edentulous maxillae in a clinical investigation. The mean angular deviation, they found, was 5.02 ± 0.19 °. Additionally, they observed that in three dimensions, the mean deviations at the implant tip and shoulder were 1.59 ± 0.07 mm and 2.05 ± 0.07 mm, respectively.

In the present study, 3D-printed overdenture group showed significantly higher rates of occlusal wear than the conventional heat polymerized overdenture group when evaluated using 3D digital analysis after twelve months, with a p value ≤ 0.001. This may be due to the reported lower hardness of 3D-printed resin material than heat polymerized material, which leads to more wear of 3D-printed teeth over time. One of the key factors influencing resistance to abrasion and wear, as well as surface roughness and microbial adherence, is the hardness of the resin denture base material [16]. 

The hardness, was significantly inferior in the 3D-printed resin than in the heat polymerized material. This finding is in agreement with Gad et al., who compared 3D-printed denture base resin with heat polymerized acrylic resin before and after thermal cycling and showed that the 3D-printed resin had the lowest hardness. This result may be due to the material composition, where 3D-printing involves the use of monomers based on acrylic esters and has relatively low double-bond conversion compared with conventional acrylic resins [17]. 

This came in agreement with a study by Abdelrahim et al., that evaluated by using 3D digital fabrication techniques, the CAD/CAM milled material, which showed a higher hardness value, followed by the conventional heat polymerized material, the lower hardness value with the 3D-printed material [18]. 

According to the results of this study, there was no statistically significant difference between the two different construction techniques of both conventional heat polymerized overdenture group and the 3D-printed overdenture group at the time of insertion (T0) regarding occlusal force distribution using Occlusense with a p value > 0.05 level of significance. With uneven occlusion between incisors, premolars, and molars in both groups. This may be due to the conventional heat polymerized overdenture group and the 3D printed overdenture group had their denture constructed using centric and eccentric records obtained by the same technique, having the same occlusal scheme and being given to the patient for the first time (T0).

The bilateral balanced occlusion criteria are normally the recommended occlusal scheme for overdentures. Occlusense divides the arch into anterior, premolar, and molar parts to display the average occlusal forces in percentages (%) for each segment of the dentition. Occlusense data analysis relies on achieving a uniform distribution of occlusal force bilaterally and anteroposteriorly [4]. 

In the present study, comparing the conventional heat polymerized overdenture group and the 3D-printed overdenture group after twelve months (T12) regarding occlusal force distribution using Occlusense showed that there was a significant difference between both groups over time. In incisors, the P value = 0.006, premolars P value = 0.001, and molars P value = 0.001. There is also an uneven distribution of force between incisors, premolars, and molars in the conventional heat polymerized overdenture group, but more even distribution of force in the 3D-printed overdenture group. This may be due to the lower modulus of elasticity (rigidity and stiffness) of 3D-printed material than in conventionally heat polymerized material. Although denture bases made with 3D-printing represent a novel approach to denture manufacturing, their flexural strength values are currently lower than those of the majority of denture base materials [19]. which explains the difference between groups over time, with more occlusal force distribution in the 3D-printed group than the conventional one. resulting from better denture material adaptation to the underlying structures of 3D-printed overdentures over time, while the conventional heat polymerized overdenture material has a high modulus of elasticity providing less force distribution to the underlying structures over time.

The flexural strength and impact strength were significantly inferior in the 3D-printed resin than in the heat polymerized material. This finding is in agreement with Gad et al., who compared 3D-printed denture base resin with heat polymerized acrylic resin before and after thermal cycling and showed that the 3D-printed resin had the lowest flexural strength and impact strength. This result may be due to the material composition, where 3D-printing involves the use of monomers based on acrylic esters and has relatively low double-bond conversion compared with conventional acrylic resins [16]. 

This came in agreement with a study by Abdelrahim et al. [18], comparing the CAD/CAM-milled material, which showed a higher flexural modulus, followed by the conventional heat polymerized material, while and a lower flexural modulus with the 3D-printed material.

The stiffness and rigidity of the material are reflected in its modulus of elasticity. A higher flexural modulus is often advantageous in clinical settings because denture base materials with high elastic moduli are more resistant to elastic deformation. Although the flexibility of the denture base is helpful in increasing the absorbed energy before fracture, the rigidity of the denture framework is a prerequisite for the ability of a denture base to equally distribute forces to the underlying structures [19]. 

Also, in agreement with a study by Prpi´c et al. [16], the CAD/CAM milled materials exhibited higher flexural strength than heat polymerized and 3D-printed acrylics, and 3D-printed acrylics have lower mechanical properties than most other denture base materials.

On the contrary, studies by Ayman [20] and Pacquet et al. [21], found that the flexural strength of heat-polymerized PMMA was higher than that of the CAD/CAM denture base material.

The results of the flexural strength and surface hardness can be interpreted in terms of the internal structures of the materials. Because polyamide material has fewer cross-linking agents, its surface hardness may be affected [22]. When compared to conventional acrylic resins, the double-bond conversion of acrylic resins used in 3D-printing removable dentures is comparatively low, which can also have an impact on mechanical qualities [23]. Additionally, during the polymerization of the CAD/CAM resins, high temperatures and inorganic fillers enhance a few mechanical qualities, such as surface hardness and flexural strength [20].

When compared to heat-polymerized resin, 3D-printed resin has lower flexural strength, elastic modulus, impact strength, and surface hardness [24]. However, studies have shown that adding different additives can improve the qualities of materials that are 3D printed. Over time, Aati et al. discovered that the quality of the interim restoration increased when ZrO2 was added to 3D-printed resin [25]. Likewise, Mubarak et al. discovered that the 3D-printed material’s tensile and flexural strengths were enhanced by the addition of 1 weight% silver-titanium dioxide nanofiller. Additionally, Chen et al. showed that a 3D-printed resin’s flexural and impact strength were enhanced by the inclusion of cellulose nanocrystals and silver nanoparticles. Finally, improvements in impact strength, flexural strength, and hardness were discovered in a recent study examining the impacts of SiO2 NPs on 3D-printed denture base resin [26]. Considering these findings, the addition of NPs appears to be a promising reinforcing strategy for 3D-printable materials.

In 3D-printed dentures, the base is manufactured first, and then the teeth are printed separately in a later step [27]. A specific bonding agent or resin that has not been polymerized and is subsequently solidified with light can be used to adhere the artificial teeth to the denture base. Either the teeth can be fused and bonded together, or they can be bonded separately. The bond between independently manufactured teeth and 3D-printed denture bases is weaker than using conventional techniques [28]. it was demonstrated that the printed dentures showed both cohesive and adhesive breakdowns of bonding agent, but the conventional dentures only showed cohesive failures. This suggests that the conventionally produced denture group had a stronger connection. Although there has been little research in this area, it appears that printed dentures typically exhibit weaker bonds. More investigation is required to determine whether this has clinical significance, and as several adhesive techniques have been proposed, a comparison of various adhesive techniques should be carried out [29].

Despite the strength of the study, there are some limitations to be discussed. First, the small sample size (*n* = 20), which may limit the generalizability of the findings to larger populations this is because of resource constraints. Second, the short evaluation period this may not give data about long term effects. However, it still allowed for preliminary conclusion and give deep insight about occlusal mechanical wear and occlusal force distribution occurred in conventional and 3D-printed two implant-retained complete mandibular overdentures. Further studies are needed including larger sample size, longer evaluation period and evaluating other mechanical characteristics of 3D-printed overdentures compared to conventional heat cured ones.

## Conclusion

Within the limitations of this study, it was shown that implant overdentures constructed by 3D-printing techniques offer a promising results in distribution of occlusal forces for achieving occlusal equilibration. However, in term of wear resistance more developments need to be done to improve material properties.

## Electronic supplementary material

Below is the link to the electronic supplementary material.

Supplementary Material 1

Supplementary Material 2

Supplementary Material 3

## Data Availability

The datasets analyzed during the current study are available from the corresponding author on reasonable request.
