# Supplementary material for: Digital versus conventional techniques for construction of mandibular implant retained overdenture
Source: BMC Oral Health. 2025 May 5;25:686. doi: 10.1186/s12903-025-05918-2 (PMC12054292; doi:10.1186/s12903-025-05918-2)
Supplement: Supplementary file 1 — Supplementary Material 1 [file 12903_2025_5918_MOESM1_ESM.docx]

**Demographic data**

| **variable** | **Group** | **All participants (20)** | **Conventional group (10)** | **3d printed group (10)** |
| --- | --- | --- | --- | --- |
| **Age** | **40-50** | **1** | **0** | **1** |
|  | **50-60** | **14** | **8** | **6** |
|  | **60-70** | **5** | **2** | **3** |
| **Sex** | **Male** | **14** | **8** | **6** |
|  | **Female** | **6** | **2** | **4** |
| **Bone quality** | **D1** | **2** | **0** | **2** |
|  | **D2** | **7** | **5** | **2** |
|  | **D3** | **8** | **4** | **4** |
|  | **D4** | **3** | **1** | **2** |
| **Smoking** | **Smoker** | **4** | **2** | **2** |
|  | **Non-Smoker** | **16** | **8** | **8** |
