# Supplementary figures and images for: Digital versus conventional techniques for construction of mandibular implant retained overdenture
Source: BMC Oral Health. 2025 May 5;25:686. doi: 10.1186/s12903-025-05918-2 (PMC12054292; doi:10.1186/s12903-025-05918-2)

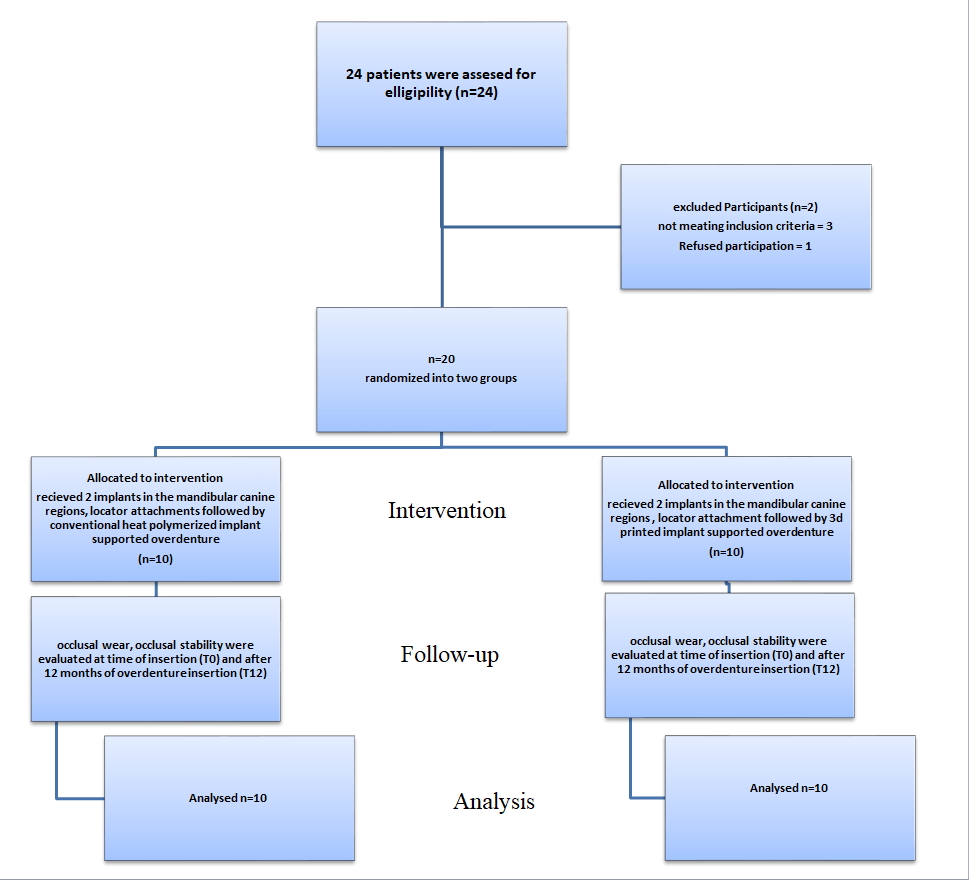

Supplement: Supplementary file 2 — Supplementary Material 2 [file 12903_2025_5918_MOESM2_ESM.png]
