# Supplementary material for: Digital versus conventional techniques for construction of mandibular implant retained overdenture
Source: BMC Oral Health. 2025 May 5;25:686. doi: 10.1186/s12903-025-05918-2 (PMC12054292; doi:10.1186/s12903-025-05918-2)
Supplement: Supplementary file 3 — Supplementary Material 3 [file 12903_2025_5918_MOESM3_ESM.docx]

**Mansoura university**

**Faculty of dentistry**

**Department of prosthodontics**

**[Informed Consent form for clinical trial]**

For participants in clinical trial (**Digital Versus Conventional Techniques for Construction of Mandibular Implant Retained Overdenture )**

**PART I: Information Sheet**

I am the principal investigator, working for prosthodontic department, faculty of dentistry at Mansoura university. We are doing research on implant overdenture for rehabilitation of edentulous mandible (conventional and 3d-printed). I am going to give you information and invite you to be part of this research.

**Purpose of the research**

The aim of this study is to evaluate and compare two different construction techniques (conventional and 3D-printed techniques) for two implant-retained complete mandibular overdentures regarding mechanical wear of the occlusal surface using 3D digital analysis and occlusion force distribution using the digital occlusal analysis system (Occlusense).

**Type of Research Intervention**

This research will involve implant placement and construction of 2 implants retained overdenture (conventional, 3d-printed) using locator attachment

**Participant selection**

We are inviting edentulous participants with the main complaint for is insufficient retention and stability of the mandibular dentures.

**Voluntary Participation**

Your participation in this research is entirely voluntary.

**Procedures and Protocol**

Two implants will be inserted into the mandible in the canine regions. After osseointegration, locator attachment will be screwed to implants. Each patient randomly will receive only one of the following overdentures : (1) conventional heat-cured acrylic resin overdenture and (2) 3D-printed overdenture. mechanical wear of the occlusal surface will be evaluated using 3D digital analysis and occlusal force distribution using a digital occlusal analysis system (Occlusense).. Evaluation will be performed immediately and after 12 months of using overdenture.

It will be necessary for you to come to the department of prosthodontics, faculty of dentistry, Mansoura university for periodic follow up visits.

**Side Effects**

Some possible side effects related to the surgical procedures include temporary swelling, pain, infection, nerve injury, implant fracture.

**Benefits**

If you participate in this research, you will have the following benefits: rehabilitation of edentulous mandible with implant supported overdenture.

**PART II: Certificate of Consent**

**I have read the foregoing information, or it has been read to me. I have had the opportunity to ask questions about it and any questions that I have asked and have been answered to my satisfaction. I consent voluntarily to participate as a participant in this research.**

**Print Name of Participant__________________**

**Signature of Participant ___________________**

**Date ___________________________**

**Day/month/year**

**If illiterate**

**I have witnessed the accurate reading of the consent form to the potential participant, and the individual has had the opportunity to ask questions. I confirm that the individual has given consent freely.**

**Print name of witness_____________________ AND Thumb print of participant**

**Signature of witness ______________________**

**Date ________________________**

**Day/month/year**

**Statement by the researcher/person taking consent**

**I have accurately read out the information sheet to the potential participant, and to the best of my ability made sure that the participant understands that the following will be done:**

**1. Surgical procedures (implant placement)**

**2. Prosthetic procedures (conventional,3d-printed overdenture)**

**3. evaluation of mechanical wear of the occlusal surface (digital occlusal analysis), and occlusal force distribution ( occlusense)**

**4. Follow up, periodic recall.**

**I confirm that the participant was given an opportunity to ask questions about the study, and all the questions asked by the participant have been answered correctly and to the best of my ability. I confirm that the individual has not been coerced into giving consent, and the consent has been given freely and voluntarily.**

**Print Name of Researcher****/person taking the consent________________________**

**Signature of Researcher /person taking the consent__________________________**

**Date ___________________________**

**Day/month/year**
